# Supplementary material for: From sequence to enzyme mechanism using multi-label machine learning
Source: BMC Bioinformatics. 2014 May 19;15:150. doi: 10.1186/1471-2105-15-150 (PMC4229970; doi:10.1186/1471-2105-15-150)
Supplement: Additional file 2 — Java code of ml2db. Additional file ml2db_code.tar.gz contains the Java source code to run the multi-label machine learning experiments and save the results to database. The code’s Javadoc is included. [file 1471-2105-15-150-S2.zip › additional file 2/ml2db/utils/doc/uk/ac/standrews/utils/main/webutils/simpledomparser/class-use/XmlNode.html]

Uses of Class uk.ac.standrews.utils.main.webutils.simpledomparser.XmlNode


JavaScript is disabled on your browser.


- Overview
- Package
- Class
- Use
- Tree
- Deprecated
- Index
- Help

- Prev
- Next

- Frames
- No Frames

- All Classes

## Uses of Class uk.ac.standrews.utils.main.webutils.simpledomparser.XmlNode

- Packages that use XmlNode

  | Package | Description |
  | --- | --- |
  |  |  |
  | --- | --- |
  | uk.ac.standrews.utils.main |  |
  | uk.ac.standrews.utils.main.webutils |  |
  | uk.ac.standrews.utils.main.webutils.simpledomparser |  |
  | uk.ac.standrews.utils.test |  |
- - ### Uses of XmlNode in uk.ac.standrews.utils.main

    Methods in uk.ac.standrews.utils.main that return XmlNode

    | Modifier and Type | Method and Description |
    | --- | --- |
    |  |  |
    | --- | --- |
    | `static XmlNode` | EntrezUtils.`getESummaryXML(java.lang.String db, java.lang.String id)` Gets an XML tree of elements (SimpleElement) from eSummary Entrez service. |

    Methods in uk.ac.standrews.utils.main with parameters of type XmlNode

    | Modifier and Type | Method and Description |
    | --- | --- |
    |  |  |
    | --- | --- |
    | `static java.lang.String` | EntrezUtils.`eSummaryToString(XmlNode xmlTree)` Test method to print an an Entrez eSummary xml report |
    | `static java.lang.String` | EntrezUtils.`xmlElementToString(XmlNode xml)` Test method to print to string a single Entrez eSummary xml element |
  - ### Uses of XmlNode in uk.ac.standrews.utils.main.webutils

    Methods in uk.ac.standrews.utils.main.webutils that return XmlNode

    | Modifier and Type | Method and Description |
    | --- | --- |
    |  |  |
    | --- | --- |
    | `static XmlNode` | XMLUtils.`getXmlFromHTML(java.lang.String url, java.lang.String xhtmlFile)` |
    | `static XmlNode` | XMLUtils.`getXmlFromReader(java.io.BufferedReader reader)` Get the XML as SimpleElement from a URL reader. |
  - ### Uses of XmlNode in uk.ac.standrews.utils.main.webutils.simpledomparser

    Methods in uk.ac.standrews.utils.main.webutils.simpledomparser that return XmlNode

    | Modifier and Type | Method and Description |
    | --- | --- |
    |  |  |
    | --- | --- |
    | `XmlNode` | XmlNode.`addChildNode(XmlNode element)` Adds a child element to the node, and returns the node itself |
    | `static XmlNode` | XmlSearcher.`depthFirstSearch(XmlNode xmlTree, java.lang.String searchString, java.lang.String attributeName, java.io.File log)` Searches this tree in a depth first mode and returns the first node whose tag, text or attribute value matches the given string. |
    | `static XmlNode` | XmlSearcher.`depthFirstSearch(XmlNode xmlTree, java.lang.String searchString, java.lang.String attributeName, java.io.File logFile, int emergencyBrake, int maxNumberOfChildren)` Searches this tree in a depth first mode and returns the first node whose tag, text or attribute value matches the given string. |
    | `static XmlNode` | XmlSearcher.`depthFirstSearchOfParent(XmlNode tree, java.lang.String desiredTag, java.lang.String attributeName)` |
    | `static XmlNode` | XmlSearcher.`depthFirstSearchOfParent(XmlNode xmlTree, java.lang.String searchString, java.lang.String attributeName, java.io.File logFile, int emergencyBrake, int maxNumberOfChildren)` TODO duplicated code! depthFirstSearch ~ depthFirstSearchOfParent Searches this tree in a depth first mode and returns the \*parent\* of the first node whose tag, text or attribute value matches the given string. |
    | `static XmlNode` | XmlNode.`getXmlTreeFromString(java.lang.String XMLText)` |
    | `static XmlNode` | XmlNode.`getXmlTreeFromUrl(java.lang.String url, boolean gzipped)` get XML tree from URL |
    | `XmlNode` | SimpleDOMParser.`parse(java.io.Reader reader)` |
    | `XmlNode` | XmlNode.`removeChildNode(XmlNode node)` Remove a child element |

    Methods in uk.ac.standrews.utils.main.webutils.simpledomparser that return types with arguments of type XmlNode

    | Modifier and Type | Method and Description |
    | --- | --- |
    |  |  |
    | --- | --- |
    | `java.util.LinkedList<XmlNode>` | XmlNode.`getChildElements()` public Object[] getChildElements() { return m\_childElements.toArray(); } |
    | `static java.util.LinkedList<XmlNode>` | XmlSearcher.`getInternalBlocks(java.lang.String externalBlockTag, XmlNode xmlTree, java.io.File log)` Gets the children of a given xml tag |

    Methods in uk.ac.standrews.utils.main.webutils.simpledomparser with parameters of type XmlNode

    | Modifier and Type | Method and Description |
    | --- | --- |
    |  |  |
    | --- | --- |
    | `XmlNode` | XmlNode.`addChildNode(XmlNode element)` Adds a child element to the node, and returns the node itself |
    | `static XmlNode` | XmlSearcher.`depthFirstSearch(XmlNode xmlTree, java.lang.String searchString, java.lang.String attributeName, java.io.File log)` Searches this tree in a depth first mode and returns the first node whose tag, text or attribute value matches the given string. |
    | `static XmlNode` | XmlSearcher.`depthFirstSearch(XmlNode xmlTree, java.lang.String searchString, java.lang.String attributeName, java.io.File logFile, int emergencyBrake, int maxNumberOfChildren)` Searches this tree in a depth first mode and returns the first node whose tag, text or attribute value matches the given string. |
    | `static XmlNode` | XmlSearcher.`depthFirstSearchOfParent(XmlNode tree, java.lang.String desiredTag, java.lang.String attributeName)` |
    | `static XmlNode` | XmlSearcher.`depthFirstSearchOfParent(XmlNode xmlTree, java.lang.String searchString, java.lang.String attributeName, java.io.File logFile, int emergencyBrake, int maxNumberOfChildren)` TODO duplicated code! depthFirstSearch ~ depthFirstSearchOfParent Searches this tree in a depth first mode and returns the \*parent\* of the first node whose tag, text or attribute value matches the given string. |
    | `static boolean` | XmlSearcher.`evaluateCondition(XmlNode element, java.lang.String wantedString)` Check whether the desired string is either a tag or text for this element. |
    | `static boolean` | XmlSearcher.`evaluateCondition(XmlNode element, java.lang.String wantedString, java.lang.String attributeName)` Check whether the desired string is either a tag, text or an attribute \*value\* for this element. |
    | `static java.util.LinkedList<XmlNode>` | XmlSearcher.`getInternalBlocks(java.lang.String externalBlockTag, XmlNode xmlTree, java.io.File log)` Gets the children of a given xml tag |
    | `static java.lang.String` | XmlSearcher.`getTextFromChildElement(XmlNode xmlTree, java.lang.String searchString, java.lang.String attributeName, java.io.File log)` Checks if child exists and returns its text |
    | `XmlNode` | XmlNode.`removeChildNode(XmlNode node)` Remove a child element |
  - ### Uses of XmlNode in uk.ac.standrews.utils.test

    Methods in uk.ac.standrews.utils.test that return XmlNode

    | Modifier and Type | Method and Description |
    | --- | --- |
    |  |  |
    | --- | --- |
    | `static XmlNode` | XmlNodeTest.`getNumberedXmlTree()` |
    | `static XmlNode` | XmlNodeTest.`getPdbSimpleElement()` |
    | `static XmlNode` | SimpleDOMParserTest.`parseAndGetSimpleElementTest()` |

- Overview
- Package
- Class
- Use
- Tree
- Deprecated
- Index
- Help

- Prev
- Next

- Frames
- No Frames

- All Classes
